# Swinepox virus: an unusual outbreak in free-range pig farms in Sicily (Italy)

**DOI:** 10.1186/s40813-024-00376-8

**Published:** 2024-07-25

**Authors:** Vincenzo Di Marco Lo Presti, Dorotea Ippolito, Giusy Cardeti, Antonella Cersini, Luigi Bertolotti, Benedetta Amato, Barbara Colitti, Chiara Giudice, Flavia Pruiti Ciarello, Domenico Vicari, Maria Teresa Scicluna, Maria Teresa Capucchio, Rosita Calogero, Michele Fiasconaro

**Affiliations:** 1 Istituto Zooprofilattico Sperimentale della Sicilia “A. Mirri”, Via Gino Marinuzzi 3, 90129 Palermo, Italy; 2 Department of Food Safety, Nutrition and Veterinary Public Health, Unit of Emerging Zoonoses, Istituto Superiore di Sanità, Viale Regina Elena, 299, Roma, 00161 Italy; 3 Istituto Zooprofilattico Sperimentale del Lazio e della Toscana “M. Aleandri”, Via Appia Nuova, Roma, 1411 – 00178 Italy; 4 Department of Veterinary Science, University of Turin, Largo P. Braccini 2, Grugliasco, Torino, 10095 Italy; 5 Department of Veterinary Pathology, Bristol Veterinary School, University of Bristol, Bristol, UK; 6 Department of Veterinary Medicine and Animal Sciences, University of Milan, Via dell’Università, 6, Lodi, 26900 Italy

**Keywords:** Swinepox virus, Swine, Poxvirus, Skin lesions, *Haematopinus suis*, biosecurity

## Abstract

**Background:**

Two outbreaks of swinepox were investigated in free-range domestic pig farms located in the northeastern side of Sicily, Italy. The disease is generally self-limiting with a low mortality rate, but morbidity can reach high rates in case of poor sanitary conditions, improper husbandry practices and ectoparasitic infestation. The presented cases are the first ever reported on the island and part of the few cases reported in domestic pigs.

**Case presentation:**

Carcasses condemned at the slaughterhouse and deceased pigs from Farm A and Farm B respectively, were referred for post-mortem examination and further investigations, with a strong suspect of SwinePox virus (SWPV) infection. Twelve deceased pigs were examined in total, showing poor body condition and pustular lesions scattered all over the cutaneous surfaces. Moreover, pigs from Farm B showed ocular lesions classified from Grade I to IV (from mild conjunctivitis to severe keratoconjunctivitis with corneal oedema, opacity, and ulcers). Final diagnosis was pursued by the microscopic assessment of skin lesions in both farms, which revealed the typical SWPV-lesion appearance, such as severe and disseminated ulcerative dermatitis and suspected inclusion bodies multifocally observed in the epidermis. Moreover, negative staining Electron Microscopy (nsEM) was performed on skin lesions and ocular swabs from Farm B, revealing in two samples the presence of brick-shaped viral particles, 220 nm long and 160 nm wide, with irregularly arranged surface tubules, identified as SWPV. The gene encoding the 482-bp fragment of the virus late transcription factor–3 was detected by PCR and sequencing revealed 99.79% identity and 100% query-cover with a strain previously isolated in Germany. Field clinical assessment was then performed in Farm B, revealing high overcrowding, poor sanitary conditions and improper husbandry practices, which are relevant risk factors for SWPV transmission.

**Conclusions:**

The present is the first case report of SWPV in free-range pigs raised in Sicily, an island of the Southern coast of Italy, and wants to raise awareness on a neglected disease, and cause of animal health and welfare issues.

## Background

Swinepox virus (SWPV) is the causative agent of Swinepox, a typical smallpox-like disease affecting pigs. This enveloped brick-shaped, double-stranded DNA virus is the only member of the *Suipoxvirus* genus, belonging to the Chordopoxvirinae subfamily that is part of the Poxviridae family [1]. Poxviruses are generally species-specific viruses [2], but spill-over events of certain species have been long-known [3]. The recent zoonotic emergence of the Monkeypox virus has raised global concerns about a possible new pandemic, reviving interest in this viral family [4, 5]. According to the current scientific literature, SWPV infects only domestic pigs and occasionally wild boars [6]. The disease usually occurs with epidemic features characterized by high morbidity [7] and low mortality rates [8, 9]. The specific tropism of SWPV for keratinocytes [10] determines multifocal to diffuse eruptive dermatitis and eventual pyodermitis due to secondary bacterial colonization [11].

The viral cycle depends on transmission events that occur through direct contact with scabs or oral and nasal secretions of infected animals. The virus robustness and the prolonged survival period in scabs (up to one year) contribute to long-term infections and disease maintenance within a given animal group. An additional transmission route described for SWPV is the vertical route. Usually, congenitally infected piglets die shortly after birth [12–14]. Furthermore, SWPV can be also vector-borne transmitted, as many arthropods, including the pig louse (*Haematopinus suis*) [15] and sporadically houseflies (*Musca domestica*) [16] may act as mechanical vectors. On the other hand, the virus has been demonstrated as a promising vector for viral vector-based vaccines [17] for the prophylaxis of many porcine and non-porcine diseases [18–23].

Swinepox has been reported in worldwide [6–8, 24, 25] even in recent times, with the latest report published in 2024 [5]. Overall, the occurrence of the disease is related to poor husbandry and poor sanitary conditions [8, 26], which are rarely encountered in modern, intensive production systems. However, the disease deeply influences pig welfare, growth rates and carcass condemnation at the abattoir. In Italy, SWPV circulation has been demonstrated in pigs in the Northern Regions since 2002 [27] and in Central Italy [28], and recently in wild boars [29]. No reports are currently available for other Italian regions, and the disease has never been notified before in Sicily, the southernmost Italian island in the Mediterranean Sea. The present report aims to describe the first-ever outbreaks of SWPV in Sicily, providing clinical-pathological and molecular features of the SWPV strain involved in two free-range pig farms.

## Case presentation

The outbreaks of SWPV arose in two free-range Nebrodi Black Pig farms, located in the Nebrodi Natural Park, the largest natural reserve in the north-east of Sicily. The Park is mostly hilly and mountainous, where wood and undergrowth give shelter to both wildlife and livestock raised in feral and semi-feral conditions. The farming system is based on outdoor farms where human intervention is limited to provide food and water supply if natural resources are not available. The distinct production phases (breeders, post-weaning and fattening pigs) are usually managed in large outdoor enclosures separated one from another according to each animal category bred.

The two SWPV outbreaks occurred in 2019 (Farm A) and in 2021 (Farm B). The first outbreak (Farm A) was notified to the Department of Territorial Assistance of the Istituto Zooprofilattico Sperimentale della Sicilia (IZSSI) - Area Barcellona P.G. after the incidental finding of SWPV-like skin lesions at the abattoir, whereas the Farm B outbreak was referred after spontaneous deaths reported in animals showing SWPV-like lesions.

### Pathological and histological examinations

**Fig. 1:**
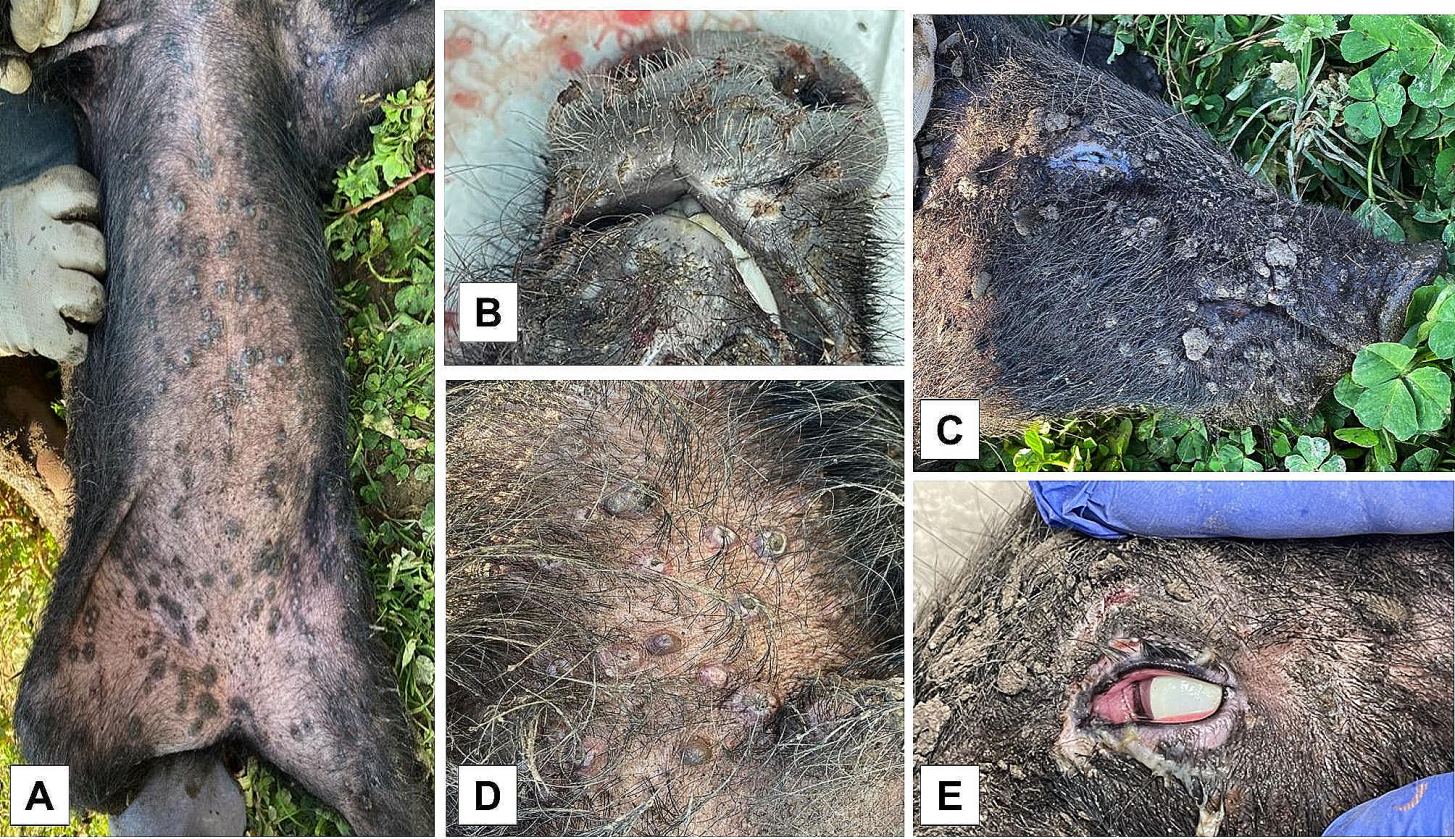
Multifocal, eruptive dermatitis caused by SWPV in Farm B. Multifocal distribution of skin lesions in the abdomen and inner surface of the limbs **(A)**, peri-labial area **(B)** and periocular area **(C)**. Lesions evolved from macules to papules and pustules with a central umbilicated area **(D)**. Severe pustular and ulcerative blepharitis with severe conjunctival hyperemia, chemosis mucous- suppurative ocular discharge and severe diffuse corneal oedema and opacity in a pig from Farm B **(E)**

A complete post-mortem examination (PME) was performed on four weaned piglets from Farm A and eight weaned piglets from Farm B, according to the standard operating procedures in use at the laboratory. Carcasses from Farm A showed severe lice infestation, whereas no parasites were detected on carcasses from Farm B. All the carcasses showed poor general conditions associated with multifocal, eruptive dermatitis. The cutaneous lesions evolved from macules or papules to pustules and umbilicated lesions, followed by erosions and crusts. The most affected areas were the ventral abdominal wall, the inner surface of the forelimbs and hindlimbs, and the periocular, peri-labial and inguinal regions (Fig. 1, A-D). Secondary pyodermatitis was also noticed. Furthermore, all carcasses from Farm B showed ocular lesions, categorized as Grade I (blepharitis) in four cases, Grade II (blepharitis and conjunctivitis) in three cases and Grade III (severe keratoconjunctivitis with corneal oedema, opacity and corneal ulcer) in one case (Fig. 1, E).

**Fig. 2:**
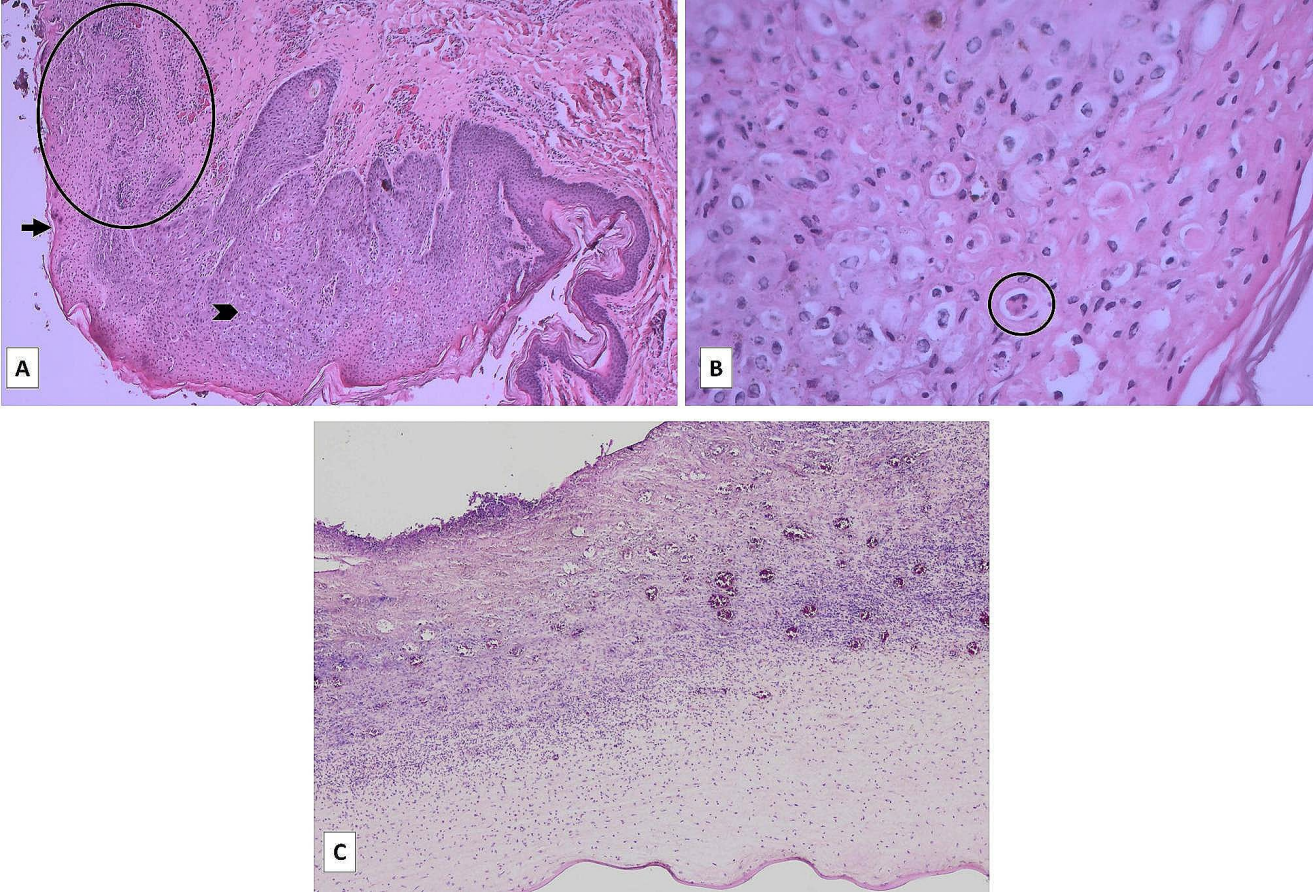
Microscopic assessment of PCR/nsEM positive skin sample **(A-B)** and eye **(C)** lesions from a Farm B affected piglet. **(A)** Severe erosion with epidermal destruction (arrow) and ballooning degeneration (arrowhead), necrosis and mixed inflammation (circled). Neoformation of blood vessels and lymphoplasmacytic infiltrates in the epidermal base was also seen. HE, 50x. **(B)** detail of the ballooning degeneration at the epidermal base with suspected inclusion bodies (circled). HE, 200x. **(C)** Severe corneal ulceration with neovascularization and mixed inflammatory infiltration of the corneal stroma. (HE,4x).

Samples of injured skin from each carcass coming from Farm A and Farm B, and affected eye globes from each animal coming from Farm B were collected in 10% buffered formaldehyde and routinely processed according to standard procedures for histopathological examination by embedding in paraffin wax, before haematoxylin–eosin (HE) staining of microtome-cut tissue sections. Histologically, the lesions showed similar features in all the samples collected from both farms, with variable degrees of severity. Due to the common histological pictures, the samples are described together. The skin showed severe and disseminated ulcerative dermatitis with parakeratotic and/or orthokeratotic hyperkeratosis, acanthosis, ballooning degeneration of epithelial cells and spongiosis. Eosinophilic, rounded, suspected inclusion bodies were multifocally observed in the epidermis. Multifocal to coalescent ulcers characterized by severe necrosis involving the epidermis and superficial dermis and composed of necrotic debris, neutrophils, fibrin, and serous proteinaceous material was also present. Hemorrhages and non-suppurative inflammatory infiltrates mainly composed of lymphocytes, plasmacells, and fewer macrophages and neutrophils with disseminated fibroblasts and new capillaries (granulation tissue) were detected at the base of the epidermis and in the dermis subjacent to the ulcerated areas (Fig. 2, A-B).

Microscopic eye assessment was performed on four out of the eight Farm B cases showing ocular lesions. In all cases, lymphoplasmacytic limbar episcleritis was present, associated with granulocytic infiltration in one case. A wide, central, crateriform corneal ulcer with necrotic debris and degenerated neutrophils was observed in three cases. In one case, the corneal epithelium showed diffuse keratosis and multifocal ballooning. Neovascularization, moderate fibrosis, oedema and multifocal to diffuse neutrophilic inflammation associated with sparse lymphocytes were recognizable in the underlying stroma in all cases. Mild, multifocal lymphoplasmacytic iritis was present in 75% of the cases, in one case, iris inflammatory infiltrate was predominantly neutrophilic, associated with hypopyon. Recent microhemorrhages and thin pre-irideal fibro-vascular membranes were also detected. Mild, multifocal to diffuse lymphoplasmacytic choroiditis was also present. The retina was severely autolytic in all the four cases (Fig. 2, C).

Additionally, during the PME, fresh skin lesions and swabs, and additional swabs of eye, nasal cavities and trachea were collected from Farm B pigs. Moreover, six formalin-fixed eye globes were also collected and sent for further analysis.

No samples were collected for virological assessment from the Farm A carcasses, as the lesions were referred to the ectoparasites, and no suspicion of SWPV infection was formulated at the time.

### Electron microscopy

**Fig. 3:**
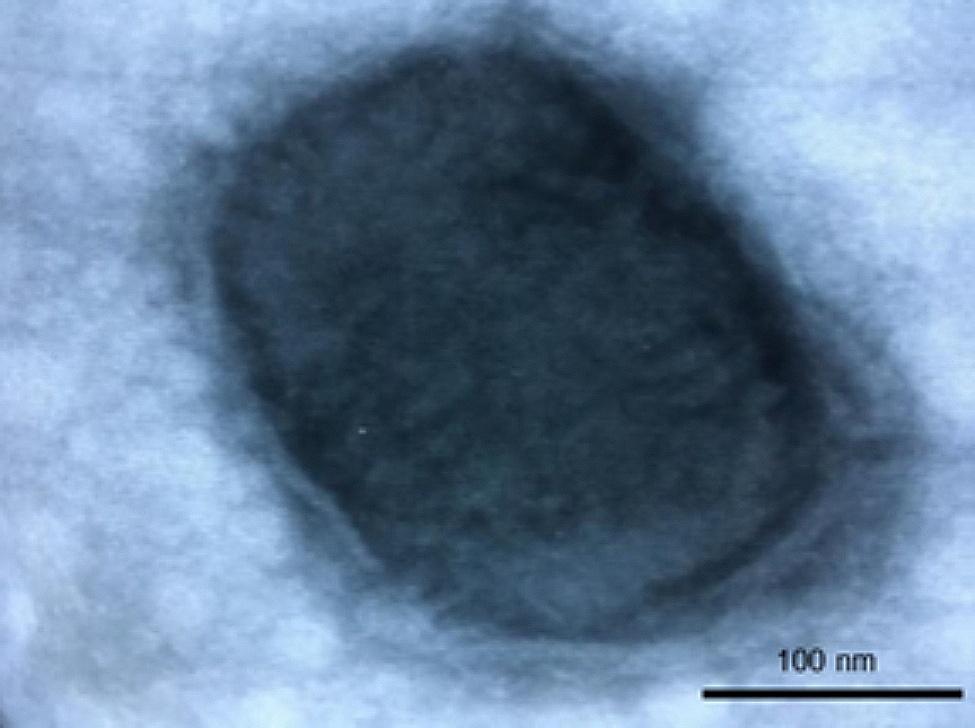
Electron micrograph. Swinepox virus particle at nsEM (2% PTA. Bar = 100 nm) performed on a eye swab coming from Farm B

Fifteen fresh samples (see Table 1) were prepared for negative staining electron microscopy (nsEM) using 2% (w/v) phosphotungstic acid stain (NaPT) (pH 6,6). Support 400 mesh copper grids, covered with a carbon-reinforced plastic film were used for the analyses. Before use, each grid was treated with Alcian Blue stain, to render the grids highly hydrophilic. The drop method (DM) was employed for fresh samples and one g of each of the two skin lesions was grinded in one ml of sterile distilled water (50% w/v). The samples were twice frozen and thawed. Each grid was first placed on a 50 µl drop of each sample for 20 min and then placed on a drop of 50 µl of 2% NaPT for 2 min for counterstaining. Different swabs (n. 13 as described in Table 1), underwent an enrichment method (EnM) and were immersed in about two ml of ultrapure water, gently shaken and pressed against the tube wall before being discarded to release the biological debris present. Each sample was subsequently clarified by centrifugation at 3,000 x *g* for 30 min at about 4 °C and then at 9,000 x *g* for 30 min. After ultracentrifugation in Airfuge Beckman^®^ for 20 min at 21 psi (82,000 x *g*), the pellet was negatively stained with 2% NaPT on the formvar-coated grid. A Philips EM 208, transmission electron microscope at x22,000 magnification at 80 kilovolts was used to observe if any virus particles were present [30] and the analysis time for each sample grid was standardized at 20 min [31]. Viral particles referring to SWPV were detected in one skin sample (Fig. 3). EM examination revealed brick-shaped particles with a length of approximately 220 nm and a width of nearly 160 nm, with irregularly arranged surface tubules. One eye swab gave a doubtful result, as the viral particles observed did not present a defined morphology in the enrichment method, while in PCR the sample presented a weak positivity. All the remaining samples were negative for any viral particles.

**Table 1:** Results of nsEM observation and molecular investigations on samples obtained from Farm B. DM: Drop Method; EnM: Enrichment Method; FFPE: Formalin-Fixed Paraffin-Embedded; *doubtful; **weakly positive; NA: not applicable

Sample	Num.	nsEM technique	Positivity	PCR positivity	Sequencing
Skin lesion	2	DM	1/2	1/2	Swinepoxvirus isolate SWPV/domestic pig/GER/2019.complete genome. Sequence identity 99,79%. Query cover 100%. Accession number MZ773481.1
Skin swab	3	EnM	0/3	0/3	NA
Eye swab	7	EnM	1*/7	1**/7	**PCR amplicon not sequenceable
Nasal swab	2	EnM	0/2	0/2	NA
Tracheal swab	1	EnM	0/1	0/1	NA
FFPE eyes	6	NA	NA	0/6	NA
**Total**	**21**	**/**	**2/15**	**2/21**	**/**

### Molecular investigations

Molecular investigations were performed on all the samples submitted to nsEM (two skin lesions and 13 swabs) and additionally on six formalin-fixed paraffin-embedded (FFPE) ocular bulbs collected during the PME of carcasses from Farm B (n. total samples = 21, **Table 1**). Nucleic acid extraction was performed using 500 µl of the homogenates and 150 mg of paraffin-embedded tissues. To deparaffinize the latter samples, FFPE ocular bulbs were treated with 10 microliters of beta-mercaptoethanol and then washed three times in 1X phosphate buffer saline solution (PBS). The first two washings consisted in the addition of 10 ml of 1X PBS, incubation with slow stirring for 15 minutes at room temperature and elimination of the 1X PBS plus paraffin at the end of each wash; instead, the 3rd washing, still consisted in the addition of 10 ml of 1X PBS, incubation at 4°C overnight and subsequent elimination of the added solution. The tissue samples and swabs were then homogenized in a plastic vial, containing a 5 mm steel bead and 800 µl of ATL Buffer, in a Tissue Lyser II (Qiagen, GmbH, Hilden, Germany) at 30 Hz for three minutes. Each homogenate sample was then subjected to centrifugation at 17,900 x *g* for 10 minutes at about 4°C. Finally, for each sample, 500 µl of supernatant was used for extraction with the automated QIAsymphony extractor and the QIASymphony Virus/Pathogen Mini kit (Qiagen, GmbH, Hilden, Germany). The extracted DNA was read using a spectrophotometer (Eppendorf Bio-photometer, ThermoFisher Scientific, Waltham, MA USA) to evaluate its concentration and the presence of impurities. The extracted nucleic acids were stored at -20°C until analysis. Amplification of the 482-bp fragment of the SWPV late transcription factor–3 [8] was performed using Ampli TaqGoldTM DNA Polymerase with Buffer II and MgCl2 (Applied Biosystems, ThermoFisher Scientific, Waltham, MA USA) and carried out in 50 µl final volume reaction with 5 µl of Buffer 10X, 3 µl (1.5 mM) of MgCl2 25 mM, 1 µl (0.2 mM) dNTPs 10 mM, 1 µl (0.6 µM) of each primer FP-A2L (5’-TAGTTTCAGAACAAGGATATG-3’) and RP-A2L (5’-TTCCCATATTAATTGATTACT-3’) (both at a concentration of 30 µM), 5 µl of the extracted DNA and 0.05 U/µl of Ampli Taq GoldTM DNA Polymerase (5 U/µl) and 33.5 µl ultrapure water. The PCR was performed using the Gene Amp^®^ PCR System 9700 (Applied Biosystems, ThermoFisher Scientific, Waltham, MA USA) and thermal profiles consisted of a cycle at 94 °C for 10 min, followed by 35 cycles of 1 min denaturation at 94 °C, 1 min annealing at 50 °C, 1 min extension at 72 °C and a final cycle at 72 °C for 7 min. The 482 bp amplicons were subjected to an automatic electrophoretic run by QIAxcell (QIAGEN, GmbH, Hilden, Germany).

The PCR products were subjected to purification with the QIAquick^®^ PCR Purification kit (QIAGEN, GmbH, Hilden, Germany) according to the manufacturer’s instructions. Subsequently the same purified PCR products were subjected to the sequencing cycle using the following reaction mixture: 10 ng of DNA template, 3 µM each primer FP-A2L and RP-A2L, 1 µl of BigDye Terminator Cycle Sequencing Reading Reaction Mix V 3.1 (Applied Biosystems, ThermoFisher Scientific, Waltham, MA USA), 1 µl of BigDye Terminator V. 3.1, 5X Sequencing Buffer and ultrapure water up to the final volume of 10 µl. Thermal profiles for the labeling reaction consisted of a cycle at 96 °C for 1 min, followed by 25 cycles of 10 s at 96 °C, 50 s at 60 °C, 4 min at 60 °C and a final cycle at 60 °C for 4 min. At the end of the sequencing cycle, unincorporated BigDye were removed using the BigDye Terminator Purification kit. The sequence obtained with the sequencer 3500 Genetic Analyzer, (Applied Biosystems, ThermoFisher Scientific, Waltham, MA USA) was recorded by the Sequencing Analysis software 7 (Applied Biosystems, Foster City, CA, USA) and analyzed by Basic Local Alignment Search Tool (BLAST) by comparing them to sequences from reference strains of different SWPV present in NCBI GenBank (http://www.ncbi.nlm.nih.gov/). Only two samples from Farm B, one skin sample and an eye swab sample of the total 21 analyzed were positive in SWPV PCR. Both positive samples were sequenced, however only for the skin sample was it possible to obtain a sequence that could be identified as SWPV with a sequence identity of 99.79% and a query-cover of 100% with the Access Number sequence MZ773481.1, SWPV isolate SWPV/domestic pig/GER/2019 (**Table 1**).

### Anamnesis and clinical investigations

Field clinical assessment was consequently performed on Farm B, during which anamnesis and relevant clinical information were recorded on live animals. Conversely, clinical assessment was not possible for Farm A, where only anamnestic data collected retrospectively were available. Animals were bred in outdoor semi-extensive open-cycle systems on both farms. At the time of the outbreak, the swine population in Farm A consisted of 200 animals (20 sows, 125 piglets, 40 post-weaning pigs and 15 fattening pigs), whilst Farm B consisted of 895 animals (129 sows, 6 boars, 7 piglets, 757 post-weaning pigs and 96 fattening pigs).

Both owners reported previous self-limiting skin lesions, which were treated with wide-spectrum antibiotics and corticosteroids, and spontaneous complete resolution in 2–3 weeks in most of the cases. In addition, recurrent lice infestation was reported, followed by routinary ectoparasiticides administration in both farms. The treatment was usually administered concurrently with Aujeszky’s disease vaccination and the castration of male piglets.

**Fig. 4:**
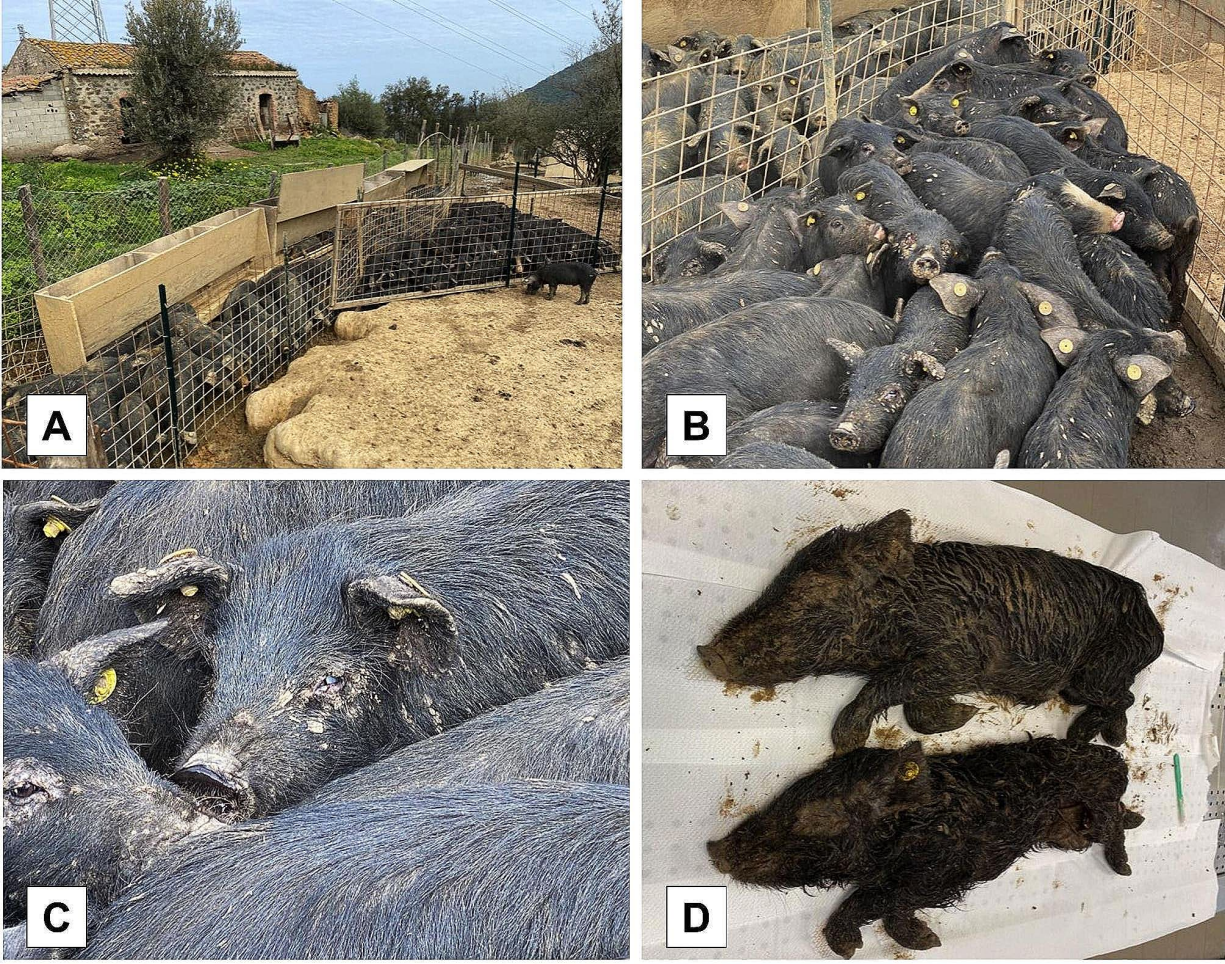
**A-B**: Lack of biosecurity measures and severe overcrowding in Farm B. **C**: Individual affected by ocular lesions. **D**: Poor body condition scores in deceased weaned pigs

In Farm B, the field assessment evidenced poor husbandry and absence of biosecurity measures, with severe overcrowding and lack of satisfactory sanitary environmental conditions (e.g. muddy surfaces and lack of dry and clean shelter areas) (Fig. 4), as well as poor animal welfare conditions. The clinical symptoms were present in 150 weaned piglets (19.81%). Clinically affected pigs showed pyrexia and poor general body conditions associated with multifocal, eruptive dermatitis and secondary pyodermatitis. The cutaneous lesions’ appearance and distribution were similar to that found in deceased animals from the same farm. No lice were detected on live animals, as the owner reported a recent anti-parasitic treatment right before the outbreak notification.

Furthermore, most of the affected animals from Farm B (106/150, 70.7%) showed ocular lesions categorized as Grade 1 in 50 cases, Grade II in 36 cases and Grade III in 20 cases.

## Discussion and conclusions

Although sporadic and self-limiting, SWPV is still reported in many countries, both in domestic and wild suids [6–8, 15, 24, 29]. When specified, the episodes refer to domestic backyard pigs [24, 26, 28], or to farms where sanitary conditions are insufficient and animal density is high [8, 26].

In the present study, the clinical assessment performed only on Farm B showed extreme overcrowding and the lack of proper sanitary interventions was the key point for the appearance of the outbreak, which affected the post-weaning sector with moderate morbidity (prevalence of 19.81%) and limited mortality rates (8 piglets). However, the farmers experienced high economic losses, as pigs were declared not fit for regular slaughtering in most cases, and most of the carcasses were destroyed.

The final diagnosis of SWPV in Farm A relied on anamnesis and direct observation of the pox-like lesions, macroscopically on carcasses and microscopically. During the post-mortem examination, only samples for histopathology were collected, as the cutaneous lesions were linked to the mechanical intervention of the detected ectoparasites. After confirmation of the outbreak in Farm B and the detection of SWPV, it was assumed that in Farm A the lesions were caused by lice which likely transmitted the virus. However, these assumptions cannot be confirmed due to the lack of laboratory data. On the contrary, in Farm B, although the virus was detected, the transmission through ectoparasites cannot be linked, as the treatment against the parasites was performed by the farmer before the outbreak notification and no lice were detected nor on carcasses or live animals.

In the presented cases, only weaned pigs were affected, which is in line to other published reports as it seems that morbidity is higher among piglets [15, 25, 26]. Another factor which could have contained the infection in the young animals is the proper separation of all the production phases in both farms (breeders, post-weaning and fattening pigs). It is also likely that stressful conditions could have predisposed juvenile pigs from both farms to be more susceptible to the disease. It is known that the post-weaning phase is one of the most delicate phase in this species, concerning stress levels [32]. Regrouping piglets from different litters to heterogeneous groups seems to be one of the main causes of stress in weaned animals. Even though a clear relationship between the effect of social relationships pre-weaning on the behavior postweaning, has not been demonstrated [33], pigs are highly sociable animals, which create groups ruled by strictly established hierarchic relationships [34, 35]. Moreover, stress can be enhanced by overcrowding, contributing to the impairment of the immune-responsiveness, animal-to-animal ectoparasites transmission and consequently vector-borne diseases diffusion, like SWPV. Another possible factor could be the concomitant administration of the antiparasitic treatment with the Aujeszky’s disease vaccination and castration, as routinely done in free-range Sicilian farms. These medical interventions require the pig to be restrained, causing relevant distress, especially in free-range pigs which may not be used to human contact, and leading to a significant impact on the immune system responsiveness of individuals [30]. In addition, stress and overcrowding can lead to an increase in aggressive behaviors, which cause wounding and possibly direct transmission of the virus. Therefore, all the abovementioned factors (stressful medical interventions, age-related susceptibility, overcrowding and traumatic events) favor the direct animal to animal spread of the virus, which, additionally, is able to persist for a long time in the environment, especially in poor sanitary conditions [36]. Indeed, SWPV transmission involves direct contact between infected and susceptible animals through wounds and biological fluids or mechanical transmission through ectoparasites, such as lice and flies [37]. As for other infectious diseases, strict biosecurity measures are mandatory to prevent the entry and spread of the infection [38].

These features are less commonly encountered in wild boars, as in the wilderness the above-mentioned risk factors are less present, not to mention the detection bias by which the disease, self-limited by nature, can resolve before leading the case can be notified. These observations might explain the sporadic nature of the reports of SWPV in wild boars [6, 29].

In the presented cases, lesions were mainly distributed in the ventral abdominal wall, the inner surface of limbs, and the periocular, peri-labial and inguinal regions, which are the election sites for ectoparasites. This is in line with what is reported in the literature for older piglets [6]. On the contrary, congenitally-infected animals show more severe skin lesions scattered all over the body and the disease is often fatal, leading to spontaneous death or euthanasia [14].

The pathological findings observed in the present study are in agreement with the classical features of the disease. Swinepox infection has overall high morbidity rates and relatively low mortality [8], due to the self-resolution of the cutaneous lesions.

However, in the present cases, a high percentage (70.7%) of animals coming from Farm B showed severe ocular lesions, which have previously been reported during Swinepox infection in pigs by Pereira et al., 2020 [39]. Ocular lesions have been reported in many Orthopoxviruses infections [40], but the exact pathogenesis is still unclear. As for Smallpox and Monkeypox, ophthalmic complications in humans include pustular eyelid rash, periorbital oedema, conjunctivitis, progressive corneal ulceration and uveitis. It has been hypothesized that one of the most likely mechanisms of ocular infection by Poxviruses is the autoinoculation of the virus from cutaneous /periocular eruption [41]. The anterior lymphoplasmacytic uveitis, in turn, could be a secondary inflammatory response to the corneal disease or could be induced by invasion of the virus into the anterior uvea, especially in immunocompromised patients. In the presented cases, bilateral ocular lesions were observed in more than 70% of the affected animals in Farm B, defining this clinical sign as one of the predominant clinical signs of the outbreak. The severity varied clinically from mild blepharoconjunctivitis, in most of cases, to severe corneal opacification and corneal ulcers. In many cases, copious ocular discharge was seen, possibly due to secondary bacterial infection. These features and their degree of spread and severity were never reported before in SWPV outbreaks. No inclusion bodies could be histologically detected within corneal epithelium, however, eye swab gave positive results to nsEM and it should be noted that in most samples the epithelium of the cornea was extensively ulcerated.

However, the skin lesion unequivocal positive both to nsEM and PCR, revealed the presence of SWPV by direct diagnosis. Eye swab, on the contrary, did not reveal a strong positivity for the virus, probably due to the low viral burden of the samples, which can explain the low positivity obtained. Moreover, the small number of samples analyzed could have impacted on the low positivity rates both at the molecular and microscopic assessments. Unfortunately, no sampling was performed on live animals due to unfavorable field conditions. Extensive and systematic sampling is therefore mandatory in order to obtain more precise and reliable information on the prevalence and magnitude of the infection in the farm.

Interestingly, the viral isolate showed a sequence identity of 99.79% and a query-cover of 100% with the strain MZ773481.1, isolated from domestic pigs in Germany and in a wild boar in Italy [6, 29]. This strain belongs to the European–North American lineage, as per the recent classification proposed by Kumar et al. [42], but the limited repertoire of genes examined in the present study did not allow for a phylogenetic analysis. The scarce data on the molecular epidemiology of the virus in Europe hinders to hypothesize how the strains are circulating through territories. However, livestock trade, wildlife movement and vector adaptation could be responsible for the spread of SWPV, but the ecology of the virus is challenging to be revealed in the absence of proper surveillance.

In conclusion, the present report describes two outbreaks of SWPV in free-range pigs in an area of Sicily (the Nebrodi Park) known for its variegated epidemiological situation and the role of the autochthonous Nebrodi Black pig in the epidemiology of some infectious diseases [43]. SWPV was never reported before in the area and although sporadic and mostly neglected, the detection of pox-like lesions should be carefully considered in order to know the real impact of this virus on the pig sector. Moreover, the importance of biosecurity measures and correct husbandry practices in farms, especially if extensive free-range farms, should be stressed, aiming to animal welfare and health preservation and to avoid severe economic losses for farmers. Finally, the occurrence of other highly contagious infectious agents, such as the Asfivirus, responsible for recent outbreaks of African Swine Fever throughout Italy, has emphasized the extreme importance of biosecurity in safeguarding extensive and intensive pig farming.

Further studies are required to isolate and characterize the viral strains that may be circulating in the Nebrodi Black pigs to elucidate the phylogeny and epidemiology of this rare but highly impactful disease.

## Data Availability

No datasets were generated or analysed during the current study.

## Abbreviations

IZSLT: Istituto Zooprofilattico Sperimentale del Lazio e della Toscana
IZSSI: Istituto Zooprofilattico Sperimentale della Sicilia
NaPT: Phosphotungstic acid
nsEM: negative staining electron microscopy
PBS: Phosphate Buffered Saline
SWPV: Swinepox virus
HE: Haematoxylin–eosin
PME: Post-mortem examination
FFPE: Formalin-fixed paraffin-embedded
DM: Drop Method
EnM: Enrichment Method
